# The Influence of Age Hardening and Shot Peening on the Surface Properties of 7075 Aluminium Alloy

**DOI:** 10.3390/ma14092220

**Published:** 2021-04-26

**Authors:** Sebastjan Žagar, Boštjan Markoli, Iztok Naglič, Roman Šturm

**Affiliations:** 1 Faculty of Mechanical Engineering, Aškerčeva 6, 1000 Ljubljana, Slovenia; sebastjan.zagar@fs.uni-lj.si; 2 Faculty of Natural Sciences and Engineering, Aškerčeva 12, 1000 Ljubljana, Slovenia; bostjan.markoli@ntf.uni-lj.si (B.M.); iztok.naglic@ntf.uni-lj.si (I.N.)

**Keywords:** aluminum alloy 7075, shot peening, microhardness, residual stresses, surface roughness

## Abstract

The present study investigates the effect of shot peening (SP) on the mechanical properties and surface roughness of 7075 aluminum alloy during different stages and conditions of heat treatment. The mechanical properties were determined by measuring Vickers microhardness profiles and residual stress profiles, while the amount of alloying elements present in the solid solution of the samples under different heat treatment conditions was determined by measuring the electrical conductivity. The results show that the increase in microhardness near the SP surface and the maximum compressive residual stresses are mainly related to the content of alloying elements in the solid solution. Surface roughness increases with increasing SP Almen intensity, and samples with the highest microhardness and residual stresses have the lowest surface roughness.

## 1. Introduction

Aluminum alloy 7075 is widely used in automotive and aerospace industries due to its high strength and light weight [1]. It has attracted much attention due to its high strength, which is comparable to that of many steels. This results in ultra-high specific strength, high fracture toughness and resistance to stress corrosion cracking [2].

This aluminum alloy contains zinc, magnesium, and copper as alloying elements that mainly contribute to its excellent mechanical properties. These mechanical properties of the alloy can be achieved by appropriate heat treatment, which includes solution heat treatment followed by quenching (rapid cooling) and artificial ageing, which can be either one or two stages. During solution annealing, the alloying elements dissolve into an α_Al_ solid solution and during quenching, a supersaturated solid solution is formed. During ageing, precipitation from the supersaturated solid solution occurs and the amount of alloying elements dissolved in the solid solution decreases. Solidification in this alloy is achieved by the formation of GP (Guinier–Preston) zones and the η’-phase (fine precipitates of Zn and the Mg-rich metastable MgZn_2_ phase), while the S’-phase (Al_2_CuMg) may also occur due to the presence of copper. Over-aging leads to the formation of the η-phase (fine precipitates of Zn and the Mg-rich stable MgZn_2_ phase) and, also importantly, to a further decrease in the amount of α_Al_ alloying elements dissolved in the solid solution.

SP [3] is a surface treatment process in which a stream of hard material shots impinges on the workpiece. This creates a thin layer of compressive residual stresses near the surface of the workpiece due to plastic deformation and strain hardening of the impact area. The treatment by SP has a positive effect on the surface of the material due to the appearance of residual compressive stresses, which increase the fatigue strength of the material and prevent the formation of cracks and the propagation of microcracks already present. The presence of a residual compressive stress field will help improve the fatigue life of a component and delay the initiation and propagation of cracks [4]. Therefore, the effects of each treatment parameter must be known, such as the selection of the treatment medium, the kinetic energy of the particles and the coverage of the tracks of each sphere. SP intensity is the measurement of the kinetic energy of the shot stream (particles). It is one of the essential means of ensuring process repeatability. The energy of the shot stream is directly related to the compressive stress that is imparted into a part. Intensity can be increased by using larger media and/or increasing the velocity of the shot stream. For performing high quality SP, the coverage of the peened surface is crucial. Coverage is the measurement of the original surface area that has been obliterated by SP dimples. Coverage should never be less than 100%, as fatigue and stress corrosion cracks can develop in the non-peened area that is not encased in residual compressive stress. If coverage is specified as greater than 100%, i.e., 200% means that the processing time to achieve 100% has been increased by that factor. A coverage of 200% time would have twice the SP exposure time as 100% coverage. SP is one of the most commonly used techniques and is still a very effective, cheap and simple process compared to other technologies such as laser shock peening [5], cavitation peening [6] with low plasticity and burnishing [7].

James et al. [8] presented information regarding the residual stress profiles in aluminum and steel welds and in SP aluminum, obtained by synchrotron and neutron diffraction, where the effects of notch, pitting corrosion and welded joints on the fatigue behavior of aluminum alloy were studied. Zupanc et al. [9] investigated the effect of surface hardening by SP on the electrochemical stability and the corrosion fatigue properties of high strength aluminum alloy 7075-T651 in the corrosive environment of a chloride solution. Xie et al. [10] investigated the distribution of residual stresses and microstructure after SP of titanium alloy (TiB + TiC)/Ti-6Al-4V composite. The results showed that both compressive residual stresses and microhardness increase with the improvement of SP intensity in the surface deformation layers. It was also found that these variations are affected by both the volume percentages of the reinforcements and the SP intensities. Mhaede [11] investigated the effects of various process parameters of SP and ball burnishing on the surface layer properties, i.e., surface roughness, microhardness and compressive residual stresses, fatigue, and corrosion fatigue properties of 7075 aluminum alloy. Ball burnishing and SP-induced plastic deformation increased the surface layer hardness and resulted in increased values of compressive residual stresses. The results also showed a significant improvement in the fatigue life tested in ambient air and the corrosion fatigue life after SP. Žagar et al. [12,13,14] investigated the influence of pitting, surface roughness, microhardness, and residual stresses on various SP aluminum alloys under different heat treatment conditions. The results showed a beneficial effect of SP treatment on the induced compressive residual stresses, which delayed the development of fatigue cracks. The research results confirmed that the application of particle kinetic energy obtained with lower air pressure values ensured a defect-free surface along with significant fatigue strength of the material. The analysis also showed that particles such as Al_7_Cu_2_Fe and MgZn_2_ play an important role in pitting corrosion and that the number and size of surface pits decreased after SP. In the paper of Martin et al. [15], the effect of SP treatment on Al 7075-T651 alloy in the case of fretting fatigue with cylindrical contact was analyzed. The SP process produces a very rough surface which would be detrimental in the case of plain fatigue. They found that the residual stress field changes with the application of stress cycles, but this relaxation seems to be mainly due to plastic flow at the beginning of the test in less than 10 cycles. Trško et al. [16] studied the relationship between the SP parameters and the fatigue fracture surface character on an AW7075 aluminum alloy. Their results showed that SP with optimized parameters produces a surface layer that can change the fatigue crack propagation mechanism and improve the fatigue strength. On the other hand, the use of extensive peening parameters decreases the fatigue strength due to the formation of surface cracks and the delamination of the surface layer. SP induces plastic deformation in the surface region of a treated material, which subsequently causes work hardening of the material. In addition to the degree of plastic deformation, work hardening of aluminum also depends on the type and amount of alloying elements dissolved in the α_Al_. The alloying elements that contribute most to the mechanical properties of alloy 7075 are zinc, magnesium, and copper. Copper and magnesium are very effective in promoting work hardening, while zinc is less effective [17]. All these elements are soluble in α_Al_ and their amount in a solid solution is highest after quenching and decreases during aging.

This alloy is commonly used in the aero and space industry for parts where wear resistance is highly valued. Therefore, we focused on exploring the potential benefits of surface hardening this alloy by shot peening. Alongside this, we wanted to gain insight into the interplay between work hardening and the resulting induced precipitation, and the role of solid solution in these processes. Research has largely focused on the effect of the SP process on the peak-aged or moderately overaged condition, while information on the quenched condition and extremely overaged conditions is lacking. Since the alloying elements in the solid solution of the 7075 alloy strongly affect the properties of the surface region after SP, we decided to evaluate the microhardness, residual stresses and roughness during all stages of the precipitation heat treatment process.

## 2. Materials and Methods

### 2.1. Material Preparation of the Surface

The alloy chosen for this investigation was 7075, which was supplied as a 10 mm thick rolled sheet from which the specimens were prepared, i.e., cutting in the longitudinal (L) and transverse (T) directions from the sheet dimensions. Cutting was achieved using a machine cutter to prepare the specimens for metallographic examination. The specimens were carefully cut to avoid the overheating of the surface and the resulting undesirable microstructural changes and introduction of additional residual stresses into the surface. All specimens were then ultrasonically cleaned in ethanol and rinsed with deionized water, followed by drying in flowing cool air. There were three specimens for each tested condition. The chemical composition of the aluminum alloy is shown in Table 1.

### 2.2. Heat Treatment

Aluminum alloy 7075 was subjected to solution heat treatment at a temperature of 475 °C for two hours and then quenched in water to room temperature. One group of specimens was left in the as-quenched condition (Q), while the other three groups were additionally artificially aged at 145 °C (Q-145), 170 °C (Q-170), and 195 °C (Q-195) for 8 h. All these samples were further treated with SP under different conditions.

### 2.3. Shot Peening

The Metal Improvement Company (MIC) in Austria carried out the SP treatment, using heat-treated S170 steel balls with a diameter between 430–480 μm and a hardness value of 43–48 HRc. The alloy samples were treated with Almen intensity 4A and 8A, with coverage set at 100% and 200%. As an example of sample designation, the sample that was solution annealed at a temperature of 475 °C for two hours, then quenched and artificially aged at 145 °C for 8 h, and later treated SP with Almen intensity 4A and 200% coverage was designated as Q-145-4A-200.

### 2.4. Electrical Conductivity

**Figure 1:**
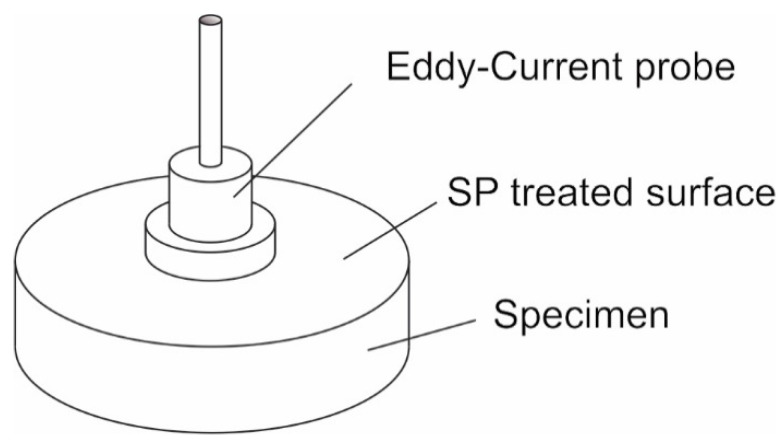
A scheme of measuring electroconductivity with eddy current probe.

The electrical conductivity of the samples was measured after quenching and after quenching followed by artificial aging at three different temperatures. By measuring the electrical conductivity of the samples, it is possible to estimate the amount of alloying elements dissolved in the α_Al_ solid solution. The electrical conductivity of the samples was measured at room temperature using Sigmatest 2.069 at 60 kHz. The electroconductivity measurements used the eddy current technique for measuring the electrical conductivity of materials based on the complex impedance of the measuring probe. The measurement was performed by placing a probe on each sample three times, which gave us information about the conductivity of the material, as seen in Figure 1. Measurement at a frequency 60 kHz and at a frequency 960 kHz was originally intended. However, since the data did not differ significantly in depth and on the surface, we decided to present the data only at a frequency of 60 kHz, which can be seen in Table 2.

### 2.5. Microhardness

Microhardness profiles in the surface layer of each sample show the influence of heat treatment and strain hardening induced by SP. Microhardness was measured using Vickers hardness at a load of 200 g (HV_0.2_). The microhardness profile started 50 μm below the surface with a step size of 25 μm for each subsequent measurement. Fourteen measurements to a depth of 375 μm were made on each individual sample, yielding reliable microhardness variation in the treated surface layer.

### 2.6. Residual Stresses

Residual stress profiles have a strong influence on the behavior of components under dynamic loading. The analysis of residual stresses using the blind hole drilling method presents the evolution of compressive residual stresses in the material under consideration and their profile as a function of depth.

The residual stresses measured in the thin surface layer were based on the relaxation blind hole drilling method according to the ASTM standard [18], using a measuring rosette CEA-06-062-UM and a pneumatic drilling turbine to achieve high drilling speeds, Vishay RS-200 [19]. The residual stress profile in the SP treated samples was determined using an integral method and the H-drill 3.10 program package. The integral method provides a separate evaluation of the residual stress at each depth increment [20]. It thus has the highest spatial resolution of all methods and is the method of choice when measuring rapidly varying residual stresses, such as SP.

### 2.7. Surface Roughness

The Characteristics of the samples selected for roughness evaluation SP are the mean arithmetic roughness R_a_ and the mean roughness depth R_z_. Numerous authors use the mentioned characteristics to describe the surface roughness in their studies on shot-peened surfaces. Both characteristics, R_a_ and R_z_, are obtained from a surface profile recorded with a Taylor–Hobson profilometer. They were calculated with the software TalyProfile Silver 7.4. The surface roughness profiles of the samples were measured at a gage length of 8 mm, with 10 repetitions on each sample. All measurements were made in different directions at the specimen edge and center. Based on the selected surface profiles and the values of the selected features, a surface finish of untreated and treated samples under different hammering conditions could be evaluated.

## 3. Results and Discussion

The aim of this work is to evaluate the effect of SP on the microhardness, residual stresses, and surface roughness of 7075 alloy at different heat treatment conditions. The microhardness and electrical conductivity of the samples in the quenched condition (Q) and in the quenched condition followed by three different artificial ageing conditions (Q- XXX) are shown in Table 2.

**Figure 2:**
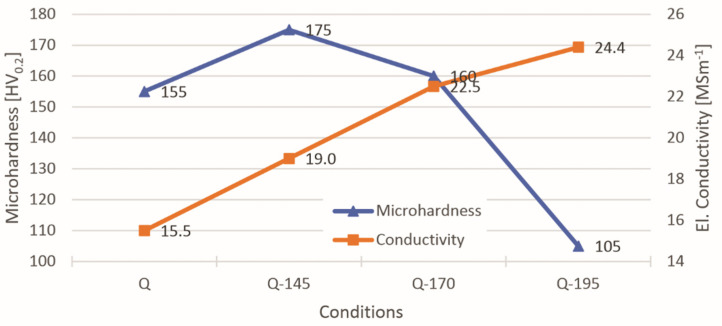
Relationship between base material microhardness and electrical conductivity for different heat treatment conditions.

It can be seen from Figure 2 that heat treatment after quenching leads to the precipitation of alloying elements, e.g., MgZn_2_. The highest microhardness is achieved after artificial ageing at 145 °C (Q-145), while higher temperatures of artificial ageing led to a decrease in microhardness due to over-ageing and at the same time, the electrical conductivity increases, as shown in Table 2 and also in Figure 2. The microhardness after quenching is only slightly lower than the microhardness of sample Q-170, but still significantly higher than that of sample Q-195. As expected, the electrical conductivity is lowest for sample Q in the as-quenched state and increases with increasing ageing temperature. This result confirms that the amount of alloying elements in the α_Al_ is highest for the unquenched sample Q and decreases with artificial ageing. The increase in the artificial ageing temperature also leads to a decrease in the amount of alloying elements in a solid solution of α_Al_.

### 3.1. Microhardness Profiles

**Figure 3:**
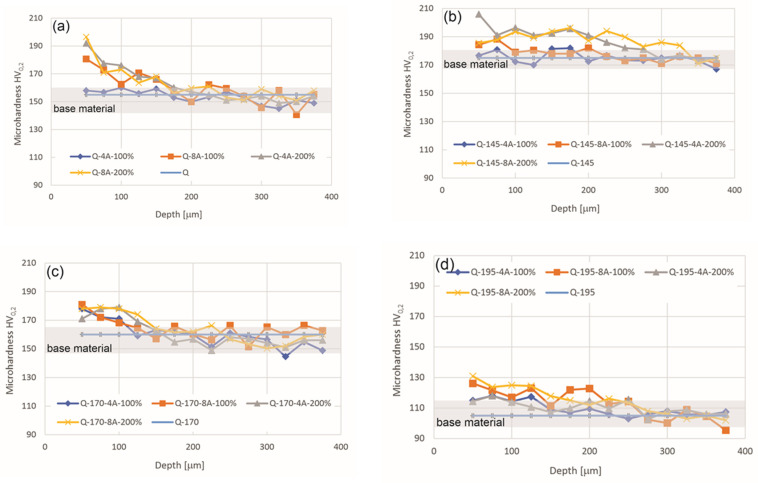
Variation of microhardness profiles in samples under different SP and ageing conditions: (**a**) in quenched condition, (**b**–**d**) after ageing at different temperatures of 145 °C, 170 °C and 195 °C, respectively.

Figure 3 shows the microhardness profiles of the as-quenched and artificially aged aluminum alloy samples after SP with the coverages of 100% and 200% and intensities of 4A and 8A compared to the microhardness of the base material, represented by a horizontal line without markings and a grey area representing the deviation from the reference value for each heat treatment condition. The microhardness profiles show that the microhardness is generally higher near the SP surface, while as the distance from the surface increases, the microhardness decreases to the value of the base material. The first depth measurement was made as close to the surface as possible, but due to the size of the imprint caused by the 200 g load, it was not possible to measure closer than a 50 μm distance. The highest microhardness 50 μm below the surface SP was obtained in the groups of specimens that were as-quenched (Q) and quenched followed by artificial ageing at 145 °C (Q-145)—Figure 3a,b—except for the specimens that were SP at 4A-100. The group of samples that were overaged (Q-170 and Q-195)—Figure 3c,d—achieved lower microhardness, especially the Q-195 group.

The increase in microhardness at 50 μm below the SP surface and the microhardness of the base material are shown in Table 3. The increase in microhardness actually corresponds to the extent of strain hardening induced by SP.

The results in Figure 3 and Table 3 show that the largest increase in microhardness due to SP was observed in sample Q-8A-200 and the lowest in sample Q-145-4A-100. The lowest increase in microhardness in sample Q-145-4A-100 was expected because the energy and coverage of SP was the lowest, while the microhardness of the base material was the highest. It is expected that it is very difficult to induce plastic deformation with a low energy of SP in a very hard material. On the other hand, sample Q-8A-200 shows the largest increase in microhardness due to the high energy and coverage of SP. Unlike the previous samples, this sample also contains the highest amount of alloying elements in the solid solution of α_Al_, which is very effective in promoting strain hardening. Sample Q-8A-200 compared with the other samples 8A-200 (different heat treatment conditions) clearly shows the effect of the high content of alloying elements in the solid solution. The extremely overaged sample group Q-195 has a very low microhardness of the base material (105 HV_0.2_), but the increase due to work hardening by SP is still small due to the low content of alloying elements in the solid solution of α_Al_.

These statements are consistent with previously published work that looked at the surface hardening of 2024 aluminum alloy when it was shot peened with a soft Zn-based ball. They found that after SP, the surface hardness of AA 2024 increased to about 140 HV from 65 HV of the base material [21].

### 3.2. Residual Stresses

**Figure 4:**
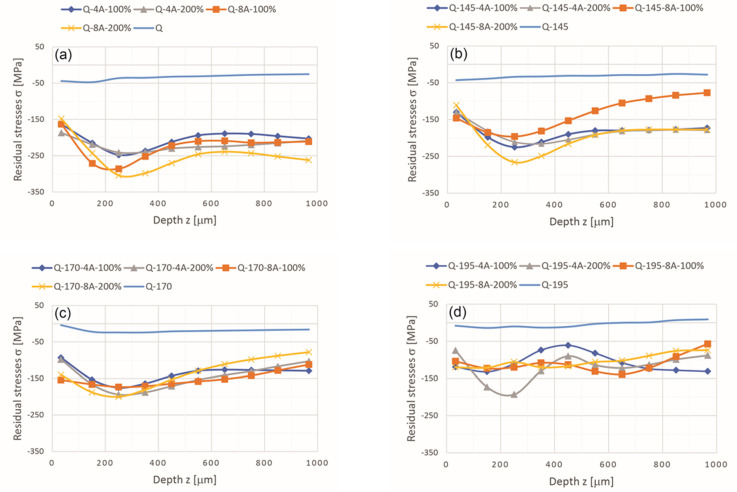
Residual stresses at different heat treatment conditions before and after SP: (**a**) in quenched condition, (**b**–**d**) after aging at different temperatures of 145 °C, 170 °C and 195 °C, respectively.

Figure 4 shows the residual stresses of the as-quenched and artificially aged specimens before and after SP under different conditions. The residual stresses before SP are predominantly compressive stresses in all four heat treatment conditions and range from (−)50 MPa in compression to (+)10 MPa in tension, depending on the heat treatment conditions.

SP increases the compressive residual stresses in all specimens. The shapes of the residual stress curves at depth are very similar for all four different SP parameters; except for the overaged samples Q-195, all other samples show the maximum compressive residual stresses at depth between 200 μm and 300 μm. It is important to note that the residual stresses obtained at depths up to 1 mm are still compressive. The highest maximum compressive residual stress was found in as-quenched sample Q-8A-200 at 304 MPa, while maximum values exceeding 250 MPa were also found in samples Q-8A-100 at 286 MPa and Q-145-8A-200 at 266 MPa. Other specimens, especially the majority of the specimens from the Q-195 group, reached much lower maximum values of compressive residual stresses between 100 MPa and 150 MPa. It is known that more intense SP induces larger compressive residual stresses. Consequently, the samples showing maximum residual compressive stresses above 250 MPa are also those showing the highest microhardness at 50 μm below the surface SP.

Similar results have been obtained by various authors describing a very similar profile and magnitude of compressive residual stresses of SP aluminum alloys [11,22,23].

### 3.3. Surface Roughness

**Figure 5:**
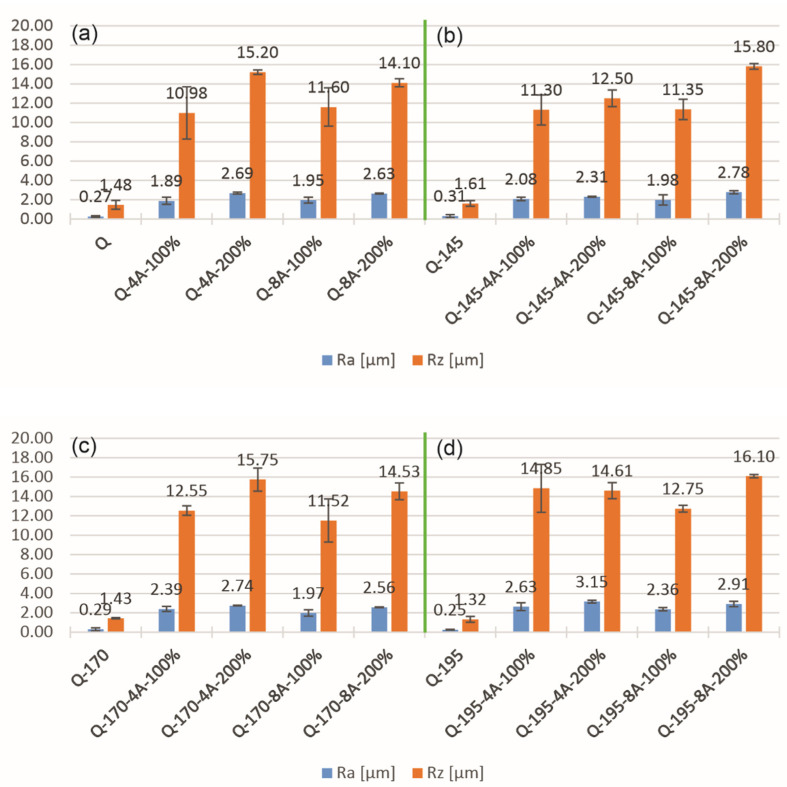
Surface roughness Ra and Rz in quenched condition (**a**) and after aging at (**b**) 145 °C, (**c**) 170 °C and (**d**) 195 °C.

Figure 5 presents two bar graphs showing the mean arithmetic roughness R_a_ and the mean roughness depth R_z_ of the surface profile before and after SP treatment of the aluminum alloy. Under all heat treatment and SP conditions, both roughness parameters, R_a_ and R_z_, significantly exceeded the values of the samples before SP. The lowest roughness R_a_ and R_z_ after SP was found in sample Q-4A-100, while the highest was found in sample Q-195-8A-200.

From the surface roughness data, it can be inferred that an increase in Almen intensity leads to an increase in surface roughness. The majority of the cases also show that 200% coverage leads to slightly lower roughness compared to 100%. Moreover, the results show that the roughness increases slightly with higher aging temperature. Aging leads to a decrease in microhardness and thus an increase in surface roughness, as a soft material deforms more easily. It is also important to mention that the overaged samples contain less alloying elements in the solid solution, which means that such a material is not as intensively work hardened during plastic deformation as the material with a higher content of alloying elements in the solid solution. The over-aging is most noticeable in the samples aged at 195 °C (Q-195), which show a much larger surface roughness, but still at a level of up to 10% increase.

Bagherifard et al. [24] also studied the surface roughness evolution induced by SP. The results obtained from numerical simulations agree quite well with the roughness values measured experimentally at SP. The roughness increases with increasing shot diameter and/or shot velocity. It also increases with increasing coverage; in which case, it tends to reach a steady state in terms of R_a_ as the process time progresses. These results also agree with our findings.

As-quenched (Q) samples and samples aged at 145 °C (Q-145) exhibit higher microhardness and maximum compressive residual stresses. These specimens also possess lower surface roughness compared to the overaged specimen groups Q-170 and Q-195. High microhardness, high maximum compressive residual stresses, and low roughness are desirable properties, rather than vice versa. The microhardness of the base material is higher for specimens Q-145, while the microhardness after SP is similar for both specimen groups. These results clearly show that, for the same conditions of SP, the specimens (Q) are hardened much more intensively than the specimens aged at 145 °C (Q-145). The more intense strain hardening is attributed to a higher content of alloying elements in the solid solution of the quenched samples which also leads to the highest residual stresses and the lowest roughness.

The experimental work presented here deals only with the “as-quenched” and “peak aged” states, while states between these two might lead to even higher microhardness after SP. There is a considerable contribution of precipitation hardening combined with a still relatively high content of alloying elements remaining in the solid solution of α_Al_ in such a state. Therefore, further work should also be directed to the aging conditions that do not correspond to the maximum microhardness values. On the other hand, the results of this work also suggest that the alloying elements dissolved in the solid solution play an important role during SP, especially elements that strongly promote strain hardening. Such elements in aluminum alloys include silicon and manganese, in addition to the aforementioned copper and magnesium [17], which means that alloys from groups 2xxx, 3xxx, 5xxx, and 6xxx could also be suitable (promising) for the application of the SP process.

## 4. Conclusions

The effect of SP on microhardness, residual stresses, and surface roughness of 7075 alloy under different heat treatment conditions was evaluated. The results show that the highest microhardness 50 μm below the surface of SP was found for the samples in quenched condition (Q) and the samples quenched and aged at 145 °C (Q-145), while the lowest was found for the samples quenched and aged at 195 °C (Q-195). The highest residual stresses were found between 0.2 and 0.3 mm below the SP surface in the samples in the as-quenched condition (Q), while the lowest were found for the samples quenched and aged at 195 °C (Q-195). Surface roughness was also found to increase after SP with an increase in Almen intensity from SP. When comparing the same Almen intensity and coverage, the highest surface roughness R_a_ and R_z_ after SP was found for samples quenched and aged at 195 °C (Q-195). The results of this work also suggest that a high content of alloying elements that promote strain hardening of aluminum, dissolved in a solid solution, results in higher microhardness and residual stresses and lower surface roughness, all of which are desirable properties. Moreover, we find it very challenging to study in detail the behavior of alloy 7075 in the as-quenched and peak-aged states after being subjected to SP, in combination with the natural ageing process.

## Figures and Tables

**Table 1:** Chemical composition of 7075 (wt.%).

Zn	Mg	Cu	Cr	Fe	Si	Mn	Ti	Al
5.70	2.36	1.28	0.19	0.17	0.12	0.05	0.03	Bal

**Table 2:** Microhardness and electrical conductivity of the samples in different heat treatment conditions before SP. All the samples were solution annealed at 475 °C for 2 h before quenching.

Designation	Microhardness before SP (HV_0,2_)	Electrical Conductivity (MSm^−1^)
Q	155	15.5
Q-145	175	19.0
Q-170	160	22.5
Q-195	105	24.4

**Table 3:** Microhardness of base material and increase in microhardness in 50 μm below the SP surface for different heat treatment and SP conditions.

Designation	Base Material Microhardness (HV_0,2_)	Difference in Microhardness (HV_0,2_)			
		4A-100	4A-200	8A-100	8A-200
Q	155	3	37	26	42
Q-145	175	2	31	9	11
Q-170	160	18	11	21	18
Q-195	105	10	9	21	26

## Data Availability

Data is contained within the article.
